# Treatment Perception and Utilization of Dental Care During Pregnancy Among Women Visiting Antenatal Clinics in King Abdulaziz Medical City & Primary Healthcare, National Guard, Jeddah, Saudi Arabia

**DOI:** 10.7759/cureus.56900

**Published:** 2024-03-25

**Authors:** Raneem Y Azab, Jawan A Binyaseen, Ahlam S Almuwallad, Shahd S Alomiri, Neda M Faden, Majed M Ramadan, Taghreed Aldosary

**Affiliations:** 1 Dentistry, Umm Al-Qura University, Makkah, SAU; 2 Dentistry, King Abdulaziz University, Jeddah, SAU; 3 Public Health, Loma Linda University, Loma Linda, USA; 4 Medical Sciences-Oral Biology, Ministry of National Guard - Health Affairs, King Abdulaziz Medical City, Jeddah, SAU

**Keywords:** dental care, jeddah, pregnant, pregnancy, dental hygiene

## Abstract

Objectives

To identify how readily accessible dental care is to a sample of pregnant women in King Abdulaziz Medical City (KAMC), Jeddah, and to determine any potential obstacles to receiving dental care while pregnant.

Methodology

Female patients visiting antenatal clinics in KAMC in Jeddah, Saudi Arabia were the target group for this cross-sectional study. The age range was limited to childbearing age (18-48 years old). Both pregnant and non-pregnant women were established in obstetrics and gynecology clinics. The pattern of dental service use and attitude toward dental treatment during pregnancy were assessed using a self-administered questionnaire. Other data were gathered, such as demographics, education, employment status, and the number of live births.

Results

This study included 361 participants in the survey with an 80% response rate. A large proportion of participants was in the age group of 19 to 35 years old (75.07%; p-value < 0.0001), holding undergraduate degrees (58.17%; p-value < 0.0001), housewives (77.56%; p-value < 0.0001), married (99.45%; p-value < 0.0001), non-pregnant women (75.07%; p-value < 0.0001), and have three or more children (42.94%; p-value < 0.0001). About two-thirds of the participants reported using private hospitals for their dental services (65.37%; p-value < 0.0001), while 22.03% (p-value < 0.0001) of the participants reported visiting a dentist in the last six months and 7.2% (p-value < 0.0001) visited a dentist during pregnancy. In terms of awareness questions, 72.02% (p-value < 0.0001) reported that if the mother did not eat well, the baby takes calcium from the mother’s teeth, 43.77% (p-value < 0.0001) reported brushing teeth at least three times a day, and 42.94% (p-value < 0.0001) of women reported that they do not have an idea about what they need to do if a pregnant woman needs treatment that requires taking X-rays. Similar patterns were observed in other awareness answers.

Conclusion

Based on the study's findings, there is a significantly low rate of dental care utilization in the sample of pregnant women. We conclude that educated women are more likely to maintain good oral hygiene and are more satisfied with their oral health. However, a large proportion of participants reported dental problems during their pregnancy. In general, a lack of knowledge about the safety of dental care during pregnancy is the main obstacle to seeking dental care.

Limitations

The selected sample was from antenatal clinics in KAMC & Primary Healthcare, National Guard, Jeddah, Saudi Arabia. As a result, the findings of this study cannot be applied to the total female population of Jeddah, Saudi Arabia. Because the information was self-reported, which is a common issue with self-administered questionnaires, and because participation in the study was voluntary and participant confidentiality was maintained, there is a low chance that the data may be subject to recall or response bias.

## Introduction

The utilization of dental services by female patients has been found to be higher than male patients [1,2]. However, different studies suggest that women do not visit dental clinics and they receive less dental care than usual when they are pregnant [3,4]. Available studies found that more than 40% of pregnant women did not visit the dentist during their pregnancy. The majority of those women utilize dental services when they are in pain only [5]. So, researchers emphasized both the importance and the safety of routine dental care for pregnant women [6]. Recent research found that women who used to visit the dentist frequently before they got pregnant are more likely to continue visiting the dentist during their pregnancy and it is found to be higher among educated pregnant women [7]. Pregnancy involves different physical and hormonal changes that might significantly affect oral health and increase the risk and severity of oral disease, especially periodontal health [8]. Teeth are considered gender-free while the supporting tissue of the periodontium is vulnerable to physiological changes, such as the levels of circulating steroid hormones in males and females [9]. Therefore, women need to understand the importance of oral health care for themselves and their children, during and after their pregnancy [10]. Several studies proved that pregnant women have a higher incidence of gingival inflammation than non-pregnant women, as the high estrogen level in pregnant women can lead to periodontal inflammation, edema, sensitivity, and the tendency to bleed easily, and it might worsen the pre-existing gingivitis if the plaque is not removed [11,12]. The increase in the severity of gingival symptoms in pregnant women reflects the microvascular physiological effect of elevated levels of estrogen and progesterone [13].

Recently, Dinas et al. and López et al. in their studies found that periodontal disease is an independent risk factor for premature birth and low birth weight, as interventional studies found that periodontal therapy can significantly reduce the rate of premature birth in women with periodontal disease [3,14]. On the contrary, studies conducted with high research quality observed that periodontal treatment does not have a positive impact on pregnancy outcomes [15]. Al-Swuailem et al. discovered that there is a lower rate of dental service utilization among the sampled pregnant women (74%) compared to non-pregnant women (84%) as regular oral hygiene measured by tooth brushing at least once a day [7]. Marchi et al. reported that 52% of women had a prenatal dental problem, with 62% not receiving care and 65% of women during pregnancy had no dental visit [4]. The lack of perception of dental needs and safety concerns are the main reasons for not visiting the dentist during pregnancy [7]. Mumcu et al. reported that utilization of dental services is influenced by different variables, such as sociodemographic characteristics of the individual, perceived dental health, people's health beliefs and attitudes toward dental problems, dental fear, and financial problems [1]. It has been proved by different studies that dentists tend to postpone the treatment during pregnancy until possible risks that may affect the mother or the fetus are eliminated [16-18]. So, the importance of dental care during and after pregnancy should be assured by both the obstetricians, as they play an important role in maintaining these dental visits, and the dental care providers, as they have a role to play in providing information about the importance of dental care to expectant mothers [19].

A study by Al-Swuailem et al. stated that there is a lack of knowledge among pregnant women in Saudi Arabia regarding the utilization of dental services, as the percentage of pregnant women who visited the dentist during pregnancy was less than 10%, according to one article published in 1994 [7]. Therefore, there is a need to continuously evaluate the pattern of seeking dental treatment among pregnant women in Saudi Arabia to recognize the barriers to seeking treatment during pregnancy [7]. Accordingly, the aim of the study is to assess the availability of dental care among a sample of pregnant women and identify possible barriers to dental care during pregnancy in King Abdulaziz Medical City (KAMC), Jeddah.

## Materials and methods

Study population

The population in this observational cross-sectional quantitative study was female patients attending KAMC, National Guard, and primary health care in Jeddah, Saudi Arabia. The participants were identified in obstetrics and gynecology clinics according to their age range, which was restricted to childbearing age (18-48 years old). Possible candidates were obtained and informed consent was obtained through phone calls and distributing surveys via SMS (short message service). Phone numbers were obtained from electronic health records over a period of one year from June 2020 to June 2021. Approval for this study was obtained from King Abdullah International Medical Research Center, Institutional Review Board (JED-21-427780-99647). The sample size was calculated using the Raosoft website with consideration of a 5% margin error, 95% confidence interval, and population of 5754 women in a period of one year from June 2020 to June 2021 and we used the best care while using the data source. The sample size was calculated to be 361. The sampling technique was a simple random technique for all pregnant and non-pregnant patients.

Questionnaire

A self-administered questionnaire validated by Al-Swuailem et al. (2014) [7] and modified in Arabic by Marchi et al. (2010) [4] was used after obtaining approval from the corresponding author. Demographic data were collected such as nationality, age, education, occupation, marital status, employment status, parity, and pregnancy status. Participants were asked about their usual dental treatment hospital as if it were governmental or private, then they were asked multiple questions about their opinion toward different clinical situations that pregnant women may face during dental treatment, such as if a procedure requires taking an X-ray would she continue with treatment, prefer to consult her physician, or take analgesics and/or antibiotic. Also, they were asked whether a pregnant woman should take local anesthesia and/or extract a tooth during pregnancy, then they were asked if they trusted dentists, healthcare providers, or both equally regarding the safety of dental care during pregnancy. Participants were asked about their usual tooth-brushing habits and if they were satisfied with their oral health.

The patients' perceptions of dental care during pregnancy were estimated by using six questions. First, they were asked whether they had visited a dentist in the six months before the current pregnancy (yes/no) and during the pregnancy (yes/no). If the patient did not visit the dentist during pregnancy, they were requested to select the key reason for not seeking dental care. Reasons for not going to the dentist were lack of insurance, financial issues, the patient does not prefer going to the dentist, the patient was too busy, the patient did not think about going to the dentist, lack of perceived need, inability to attend the clinic because of the COVID-19 pandemic, the patient thought that care was unsafe for the mother itself or the infant, and the doctor advised not to undergo dental treatment during pregnancy. In addition, patients were asked if they had experienced any dental problems during pregnancy. The questionnaire is presented in Table 1.

**Table 1:** The questionnaire

Questions	Answers								
Nationality	Non-Saudi	Saudi							
Age									
Level of education	Doctorate/PhD education	Master's education	Bachelor's education	Higher education	Secondary education	Primary education or less			
Occupation									
Marital status	Single	Married							
Pregnant	I don’t know	No	Yes						
Do you have any other children, how many (male/female)?	3 or more	2	1						
Where do you usually do your dental treatment?	Private hospital	Governmental hospital							
Do you have insurance?	No	Yes							
If a pregnant woman needs dental treatment that requires taking an X-ray (can’t proceed without an X-ray), what do you think she needs to do?	I don’t have an idea about what she needs to do, I need to consult an obstetrician/gynecologist	It’s better to take analgesics and antibiotics instead of taking X-rays during pregnancy	It’s better to take analgesics instead of taking X-rays during pregnancy	It’s better to take antibiotics instead of taking X-rays during pregnancy	I don’t see any problem with taking X-rays				
If a pregnant woman needs a dental treatment that requires anesthesia, do you think it’s better for her to take it?	I don’t know	No	Yes						
If a pregnant woman needs to extract one of her teeth, what do you think she needs to do?	I don’t have an idea about what she needs to do, I need to consult an obstetrician/gynecologist	It’s better to take analgesics and antibiotics instead of tooth extraction during pregnancy	It’s better to take analgesics instead of tooth extraction during pregnancy	It’s better to take antibiotics instead of tooth extraction during pregnancy	I don’t see any problem with tooth extraction during pregnancy				
Is there any specific time during pregnancy you think is less dangerous to the infant or the mother in dental treatment?	I don’t know	I don’t prefer doing dental treatment during pregnancy at all	During the 3^rd^ trimester	During the 2^nd ^trimester	During the 1^st^ trimester				
Who do you trust to take the advice about dental treatment during pregnancy?	The dentist	The obstetrician/gynecologist	I trust them both equally						
What are your usual habits of brushing using a toothbrush and toothpaste?	I brush my teeth at least 3 times a day	I brush my teeth once a day	I brush my teeth less frequently	I don’t brush my teeth					
Are you satisfied with your oral health?	I don’t know	Not satisfied	Somewhat not satisfied	Somewhat satisfied	Completely satisfied				
Do you think the following sentences are correct or not?	Gingival inflammation can cause low birth weight	If the mother does not eat well, the child will take calcium from his/her mother's teeth	Gingival inflammation can cause premature birth	The mother loses one tooth with each pregnancy					
Questions for pregnant women only									
If you’re pregnant, at which stage are you?	I don’t know	3^rd^ trimester	2^nd^ trimester	1^st^ trimester					
If you’re pregnant, were you planning it?	No	Yes							
Have you ever visited the dentist in the last six months of this pregnancy?	No	Yes							
Have you ever visited the dentist during this pregnancy?	No	Yes							
If you didn’t visit the dentist during this pregnancy, what was the main reason for not visiting the dentist (choose only one)?	I got advised by my obstetrician/gynecologist for not having dental treatment	I think dental treatment is not safe for the infant	I think dental treatment is not safe for the mother	I don’t need dental treatment	I didn’t think about going to the dentist	I don’t have the time to go to the dentist	I don’t like going to the dentist	Dental treatment is expensive	I don’t have insurance
Have you ever experienced any of the following dental problems during your current pregnancy?	Tooth needs extraction	Tooth caries needs filling	Gingival enlargement	Painful gingiva	Gingival bleeding	Tooth mobility	Tooth pain		

Statistical analysis

Baseline demographic characteristics and characteristics of participants were determined using descriptive analyses. Univariate analysis was conducted using chi-square χ2 and Fisher's exact tests according to the nature of the categorical variables and number of participants to describe the population and investigate the treatment perception and utilization of dental care during pregnancy among women visiting antenatal clinics in KAMC. Additionally, Cronbach’s alpha was measured to evaluate reliability and internal consistency (0.80%). All statistical tests were two-sided, and findings were considered statistically significant at p < 0.05. All analyses were conducted using SAS statistical software version 9.4 (SAS Institute Inc., Cary, NC).

## Results

Our study included 361 participants in the survey with an 80% response rate. The mean age of the sampled subjects was 31.3 (±6.08) years. The patients were predominantly Saudis (97.51%), married (99.45%), pregnant (21.05%), holding an undergraduate degree (58.17%), housewives (77.56%), and had at least one child (25.76%). Most participants reported that they did not have medical insurance (93.07%) and utilized private hospitals for their dental services (65.37%). Regarding regular oral health hygiene as measured by tooth brushing, nearly half of the participants (49.58%) have a habit of brushing their teeth at least once a day. Meanwhile, 43.77% of the participants brush their teeth three times a day. Women reported complete satisfaction with their oral health (16.34%, p < 0.0001). About three-quarters of the sampled pregnant women (n = 57) mentioned that they had at least one dental problem that could require dental intervention during their current pregnancy. Also, it was reported that only 19% of pregnant women have visited their dentist during pregnancy.

**Figure 1:**
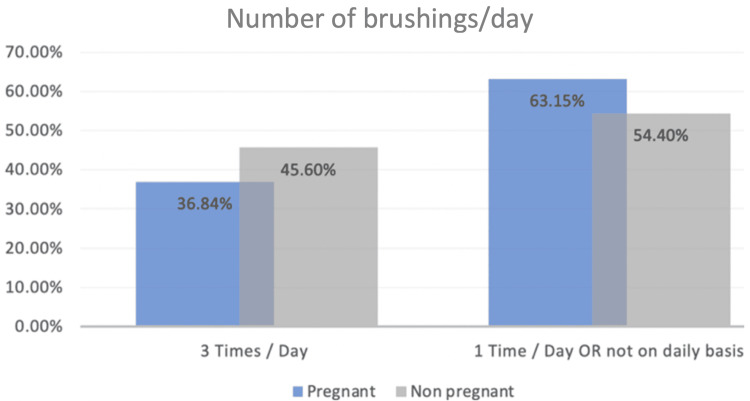
Oral habits of the sampled women

**Figure 2:**
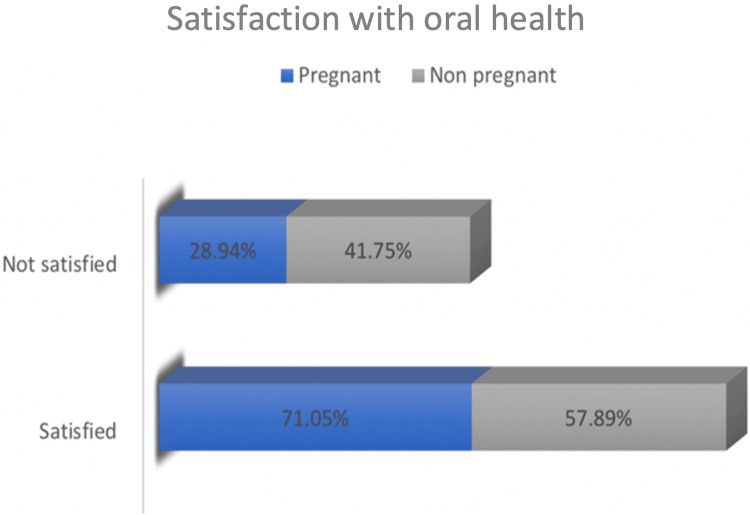
Satisfaction of the sampled women with their oral health

There was a similar pattern regarding the oral health habits among pregnant and non-pregnant women as 36.84% (n = 28) of pregnant women and 45.6% (n = 130) of non-pregnant sampled women were brushing their teeth three times a day. On the other hand, 63.15% (n = 48) of pregnant women and 54.4% (n = 155) of non-pregnant women brush their teeth once daily or not even on a daily basis (Figure 1). Regarding the satisfaction with oral health conditions, we reported that there was a higher rate of satisfaction among pregnant women when compared with the non-pregnant ones. A total of 71.05% (n = 54) of pregnant women and 57.89% (n = 165) of non-pregnant women were satisfied. While 28.94% (n = 22) of the sampled pregnant women and 41.75% (n = 119) of the non-pregnant women were not satisfied (Figure 2). The likelihood of not visiting a dentist during pregnancy was four times higher when compared to those who visited the dentist among pregnant women. It was reported that 19.33% (n = 23) of pregnant women visited the dentist during their pregnancy, while 80.67% (n = 96) did not (Table 2).

**Table 2:** Visiting a dentist during this pregnancy (for pregnant women)

Visiting a dentist during this pregnancy (for pregnant women)	Frequency	Percent
No	96	80.67
Yes	23	19.33

Approximately four-fifths of pregnant women do not seek dental care during pregnancy because of a lack of knowledge on the importance and safety of dental therapy during pregnancy. For instance, 23.68% (n = 18) of pregnant women did not go to the dentist during their pregnancy as they thought they did not need dental care, and 31.57% (n = 24) thought that dental therapy is hazardous to the growing infant and/or the mother. Approximately 10.52% (n = 8) of pregnant women who do not go to the dentist were not thinking about going while they were pregnant. About 30.2% (n = 23) of them mentioned financial barriers, which included a lack of dental insurance or the increased cost of dental procedures as a reason for not visiting the dentist. Of these pregnant women, 2.63% (n = 2) cited disliking going to the dentist, and 5.26% (n = 4) had no time for seeking dental care. Interestingly, about 1.31% (n = 1) of pregnant women who did not seek dental care reported that their doctors had advised them not to go to the dentist during pregnancy. On the other hand, as an impact of the pandemic, 13.15% (n = 10) considered COVID-19 as a barrier to visiting the dentist.

Regarding female patients' perceptions, by using the chi-square test of independence to determine whether there is a statistical difference between two categorical variables, only 11.3% (n = 41) of them agreed about exposure to X-rays during pregnancy, and the remaining 88.7% (n = 320) either preferred to consult with their physicians or take antibiotics and/or analgesics. In relation to patients' perceptions toward tooth extraction during pregnancy, 20.5% (n = 74) of them agreed. On the other hand, 36.5% (n = 132) of the participants preferred to take antibiotics and/or analgesics rather than extracting the tooth, and 42.9% (n = 155) preferred to consult their physicians first. Meanwhile, 27.9% (n = 101) of the participants accepted to receive local anesthesia when needed, and there was a significantly higher number of participants (39.6%, n = 143) who refused to receive local anesthesia during their pregnancy (Table 3).

**Table 3:** Female patients' willingness to perform dental procedures during pregnancy

Female patients' willingness to perform dental procedures during pregnancy		
Procedure	Decision	N (%)
Taking X-rays	Yes	41 (11.36%)
	No, take antibiotics and/or analgesics	162 (44.88%)
	No, consult with physicians	158 (43.77%)
Local anesthesia	Yes	101 (27.98%)
	No	143 (39.61%)
	I do not know	117 (32.41%)
Tooth extraction	Yes	74 (20.50%)
	No, take antibiotics or analgesics	132 (36.57%)
	No, consult with physicians	155 (42.94%)

According to the best time for receiving dental therapy during pregnancy, most women (pregnant and non-pregnant) were unable to ascertain that the best time for dental treatment is the second trimester. Of the sampled women, 22.1% (n = 80) do not favor performing any dental procedure during pregnancy (Table 4).

**Table 4:** Best time for receiving dental therapy during pregnancy

Timing	Frequency	Percent
During the 1st trimester	83	22.99
During the 2nd trimester	54	14.96
During the 3rd trimester	88	24.38
I don't know	56	15.51
I don't prefer doing dental treatment during pregnancy at all	80	22.16

Most participants in the study (63.1%, n = 228) generally have similar trust in gynecologists' and dentists' recommendations for dental treatment during pregnancy. Among the participants, 26.8% (n = 97) trust their obstetrician/gynecologist more than the dentist and only 9.9% (n = 36) trust their dentist (Table 5).

**Table 5:** Trust to take the advice

Trust in judgment on dental care during pregnancy	Frequency	Percent
Trust obstetricians and dentists equally	228	63.16
Dentist	36	9.97
Obstetrician/gynecologist	97	26.87

Multiple questions were asked specifically to the pregnant women about their overall oral health. Of the participants, 44.73% (n = 34) reported tooth pain, 36.84% (n = 28) reported gingival bleeding, and 21.05% (n = 16) reported gingival enlargement, with only 18.42% (n = 14) reporting painful gingival. Similar patterns were observed for tooth caries that needs filling, tooth mobility, and tooth that needs extraction at 50% (n = 38), 21.05% (n = 16), and 27.63% (n = 21), respectively.

## Discussion

This study evaluates the accessibility of pregnant women to dental clinics in Jeddah, Saudi Arabia, and identifies potential barriers during pregnancy for dental care. This research had the same findings as those reported by Al-Swuailem et al. [7] regarding the low access to dental care during pregnancy and the findings were also confirmed by other studies [3,4,16]. Compared to the results reported by Al-Swuailem et al. [7] in 2014 in Riyadh, Saudi Arabia that 22% of the sampled pregnant women visited their dentists, this study's statistics on dental care use in Jeddah suggest a similar finding, i.e., 19% of sampled women had access to dental care during pregnancy. On the other hand, the utilization of dental clinics by pregnant women in some of the regional countries is significantly higher than the findings of this study. For instance, it has been reported that the United Arab Emirates had a rate of 58%, and Kuwait had a rate of 50% of pregnant women visiting the dentist [5,17]. However, It seems that a common issue is the low level of utilization of dental services during pregnancy. Only 14% of the 351 Turkish pregnant women who were sampled for the study in 2012 went to the dentist while they were pregnant [18]. Furthermore, only 26% of a sample of 599 pregnant women used dental services on a regular basis, according to Boggess et al. [19].

The results of this study indicate that the utilization of dental services is significantly lower in pregnant women. However, the pattern of utilization of dental services is almost similar to the pattern of utilization of dental services prior to pregnancy. Fadavi et al. reported that the frequency of dental visits prior to pregnancy is a good indicator of the utilization of dental care during pregnancy [20]. This result was also confirmed by Boggess and colleagues, who stated that a woman who did not receive dental care before pregnancy has a strong prediction that she would not seek care when she is pregnant [19]. In considering one of the important aspects, the decreased utilization of dental care by pregnant women takes significant importance. Firstly, in this study's sample of pregnant women, there were higher dental needs that were reported. Approximately three-quarters (77.63%, n = 59) of the pregnant women who participated in the study stated that their current pregnancy had at least one dental issue. About three-quarters of the pregnant women in the study by Al-Swuailem et al. reported having at least one dental issue while they were currently pregnant [7]. According to Ozen et al., 69% of Turkish pregnant women who participated have reported dental issues during pregnancy [18]. Furthermore, 14.47% of the participating pregnant women do not brush their teeth on a daily basis at least once a day.

Al-Swuailem et al. reported that about 25% of the pregnant women who participated did not brush their teeth regularly on a daily basis at least once a day [7]. Ozen and colleagues found that less than 10% of pregnant women were not brushing their teeth at least once per day in their study [18]. Pregnancy is found to be correlated to an increase in gingival inflammation [10,12]. Thus, the significance of dental care during pregnancy is highlighted by these significant facts when considered in combination with the suggested possible relationship between periodontal disease and unfavorable pregnancy outcomes [15,16]. This study found that there are numerous obstacles to getting dental care while pregnant. About 8.03% of our participants reported it is not safe to have any dental treatment during pregnancy, and 9.69% of the pregnant women in the sample stated that they did not feel they needed dental care. Of the pregnant women, 5.26% reported that they do not have dental insurance, and 4.99% cited that dental treatment is expensive. Only 2.77% of our participants reported that they could not visit their dentists because of the COVID-19 pandemic. According to Dinas et al., 72% of pregnant women stated that receiving dental care while pregnant may not be safe [3]. Al-Swuailem et al. reported that almost two-thirds of the pregnant women in their sample did not perceive a need for dental care, and it is been also reported as the most common reason for not visiting the dentist in other studies as well. In addition to that, both pregnant and non-pregnant women were concerned about the safety of dental care as the primary reason for not visiting the dentist while pregnant [4,7].

Furthermore, despite all other factors, our study reported that 93.07% of our participants do not have dental insurance, and only 6.93% reported that they have dental insurance. However, Al-Swuailem et al. found that the use of dental services during pregnancy did not significantly increase because of the existence of dental insurance [7]. On the other hand, it has been found that only 14% of all pregnant women in their study had dental insurance and visited their dentist during pregnancy [18]. Other studies reported that there is a strong correlation between having dental insurance and using dental services more frequently during pregnancy [10,21]. However, Al-Swuailem et al. cited that the potential benefit of having dental insurance on the utilization of dental care during pregnancy outweighs the pregnant women's concerns regarding the safety of dental treatment. In this regard, both the dentist and the gynecologist should use scientific evidence to address pregnant women's concerns about the safety of dental treatment during pregnancy. Nevertheless, it has been discovered that pregnant women who received essential dental care or scaling and root planning in comparison to pregnant women who did not receive dental treatment during their pregnancy at 13-21 weeks of gestation had moderate to severe caries or fractured or abscessed teeth and it did not increase the risk of any unfavorable pregnancy outcomes [22]. Our findings suggested that most of the women in our sample do not have essential information regarding the safety of dental care during pregnancy as our study evidenced that the second trimester is the safest time to receive dental care, according to 19.96% of the sampled women in this study. It has been reported that the second most common reason is the financial barriers. In terms of professional trust, most of the time, the general public gave dentists a high professional trust rating [23]. However, in this study, only 9.97% of the sampled women cited that they trust dentists, and the majority of them (63.16%) stated that they equally trust their gynecologists and dentists to advise them on dental care during pregnancy. Of women who only have faith in their gynecologist or dentist, only 26.87% reported that they trust their gynecologists (26.87%).

Limitations

This study has the following limitations. First, the sample chosen was a practical sample from a single hospital; for that reason, the results of our study would not be generalizable for the entire population of women in Jeddah, Saudi Arabia. Second, the data were collected using e-surveys due to the COVID-19 pandemic precautions. However, phone calls and text messages have been utilized to get participation agreements before receiving the e-surveys and to ensure that all participants received the same e-surveys. Finally, the provided data were self-administered e-survey, as the participation was voluntary and participation confidentiality was obtained. However, there seems to be a slight tendency that the data collection will suffer from response bias.

## Conclusions

Based on the findings of this study, the following conclusions were drawn: the rate of dental service utilization is significantly lower in pregnant women; furthermore, the pattern of use of dental services closely matches the pattern of use of dental services prior to pregnancy. Nevertheless, the absence of actual need for dental treatment and safety concerns were the primary obstacles to seeking dental care during pregnancy for dental treatment and concerns regarding the safety of dental care during pregnancy. Dentists and gynecologists need to collaborate to make sure that pregnant women have adequate access to dental care. Therefore, an increase in the awareness of women in general and the pregnant ones in specific is required, to encourage them to seek regular dental visits to avoid any complicated problems and also to ask the dentist for any concerns prior to the treatment. More studies should be done in different regions of Saudi to highlight the main reasons for the reduction in the number of pregnant women visiting dentists, so more campaigns can be done throughout the country to increase awareness. The future direction of the study is to raise the idea of possibly specializing dental clinics for pregnant women only, so their concerns may decrease.
